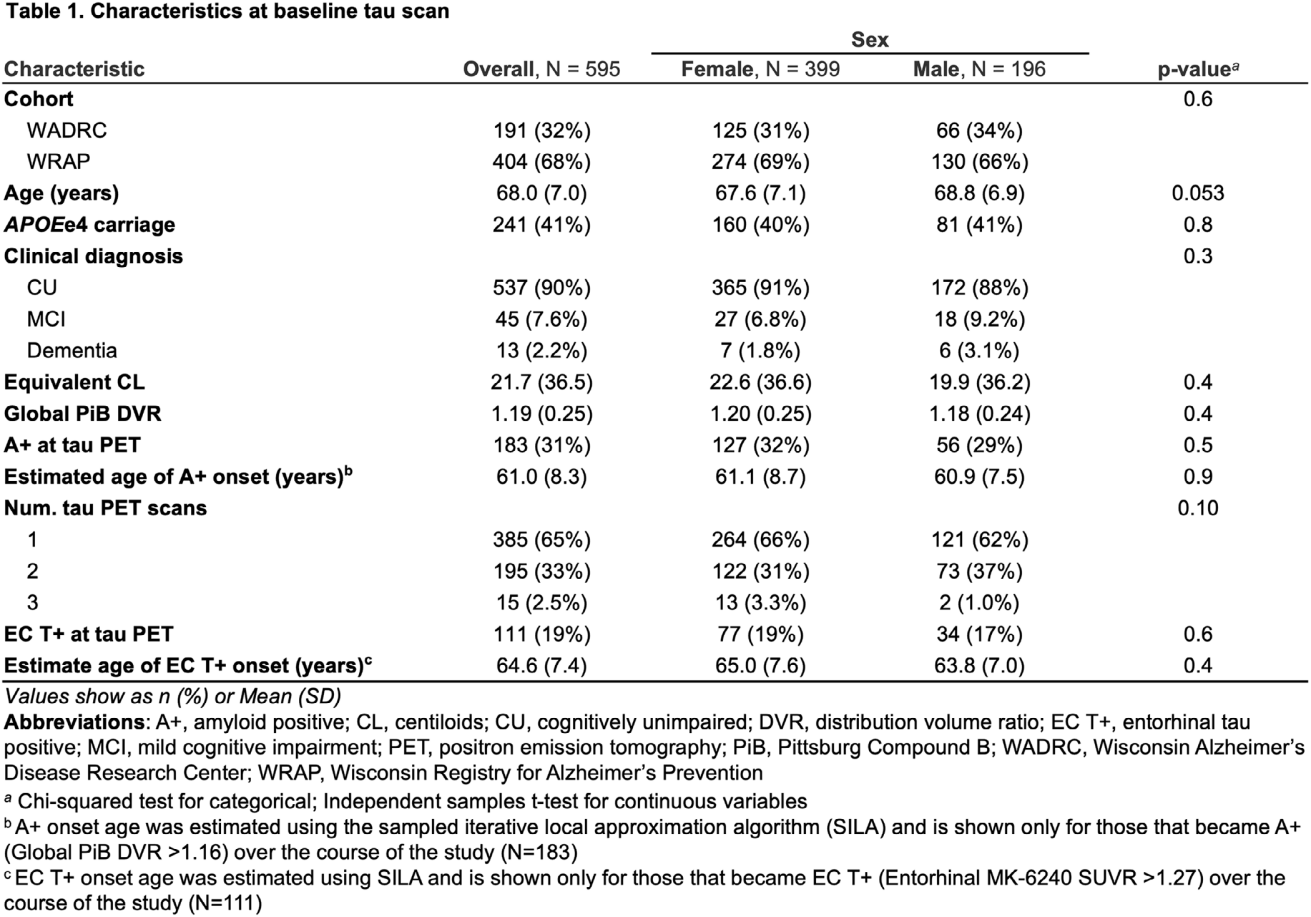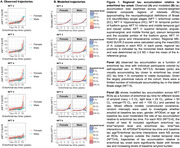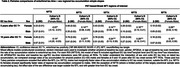# Sex and amyloid burden modify tau spatial progression following entorhinal tau onset

**DOI:** 10.1002/alz.091821

**Published:** 2025-01-09

**Authors:** Karly Cody, Margo B. Heston, Jordan P Teague, Sara Frost Alberson, Madilynn Wintlend, Melissa Bahr, Rebecca E. Langhough, Sterling C. Johnson, Tobey J. Betthauser

**Affiliations:** ^1^ University of Wisconsin‐Madison School of Medicine and Public Health, Madison, WI USA; ^2^ Stanford University, Stanford, CA USA

## Abstract

**Background:**

Characterizing factors that impact longitudinal tau progression is important for understanding tau heterogeneity observed in clinical trials and research. This work examined factors associated with longitudinal tau PET (^18^F‐MK‐6240) accumulation relative to onset of entorhinal tau (EC) to better understand neurofibrillary tangle (NFT) spatial accumulation in Alzheimer’s disease.

**Method:**

Participants (N=595; Table 1) from the Wisconsin Registry for Alzheimer’s Prevention and WADRC underwent serial amyloid and tau PET imaging. Amyloid burden was quantified from global cortical ^11^C‐PiB DVR_(0‐70)_. Tau burden was quantified in regions of interest (ROIs) corresponding to Braak NFT1‐6 using ^18^F‐MK‐6240 SUVR_(70‐90)_. Sampled iterative local approximation (SILA) was used to model EC tau trajectories and estimate EC tau‐time and onset age and, separately, to estimate amyloid level at tau scan. Regional tau accumulation was modeled with mixed effects models separately for Braak NFT2‐6 as a function of EC tau‐time (i.e. years of EC T+) starting with cubic polynomials. Models also included EC tau‐time*estimated CL at baseline tau (due to known associations between amyloid and tau) and additionally examined EC tau‐time interactions with age at baseline tau, self‐reported sex, and APOEe4.

**Result:**

In this largely unimpaired cohort, there were no significant differences observed in APOEe4 carriage, baseline clinical status or amyloid level, or estimated age of A+ or EC T+ onset across males and females (Table 1). Regional T+ followed the expected spatiotemporal pattern (NFT2‐6) as time from EC T+ onset increased (Figure 1A). Across NFT3‐6, ROIs outside the medial temporal lobe (MTL), female sex and higher baseline amyloid were each associated with significantly faster tau accumulation rates relative to EC tau‐time (Figure 1A‐B). Post‐hoc comparisons indicated that rates of tau accumulation relative to EC tau‐time increased sequentially with increasing levels of amyloid, and that in NFT3‐6, the average rate of tau accumulation was significantly faster for females compared to males (Table 2).

**Conclusion:**

These findings provide evidence that tau accumulation and spread outside the MTL is accelerated by higher levels of amyloid and female sex. These data suggest that while people follow a similar spatial progression, the rate of progression varies, which may inform tau‐related dementia prediction.